# Prognostic value of systemic immune-inflammation index in non-metastatic clear cell renal cell carcinoma with tumor thrombus

**DOI:** 10.3389/fonc.2023.1117595

**Published:** 2023-01-26

**Authors:** Yufeng Gu, Yao Fu, Xin Pan, Yulin Zhou, Changwei Ji, Tangliang Zhao, He Miao, Huichen Lv, Jianping Da, Jingping Ge, Linhui Wang, Le Qu, Silun Ge, Hongqian Guo, Wenquan Zhou

**Affiliations:** ^1^ Department of Urology, Jinling Hospital, Clinical School of Medical College, Nanjing University, Nanjing, Jiangsu, China; ^2^ Department of Pathology, Drum Tower Hospital, Clinical School of Medical College, Nanjing University, Nanjing, Jiangsu, China; ^3^ Department of Urology, Drum Tower Hospital, Clinical School of Medical College, Nanjing University, Nanjing, Jiangsu, China; ^4^ Department of Urology, Jinling Hospital, Jinling School of Clinical Medicine, Nanjing Medical University, Nanjing, Jiangsu, China; ^5^ Department of Urology, Changhai Hospital, Naval Medical University, Shanghai, China

**Keywords:** non-metastatic clear cell renal cell carcinoma, tumor thrombus, biomarker value of systemic immune-inflammation index, systemic immune inflammation index, nomogram

## Abstract

This study aims to determine the prognostic value of SII for non-metastatic clear cell renal cell carcinoma (ccRCC) patients with venous tumor thrombus (VTT). We retrospectively collected and analyzed 328 non-metastatic ccRCC patients with VTT who underwent radical nephrectomy and thrombectomy from 3 tertiary centers in China between 2011 to 2021. Kaplan-Meier analyses and Cox proportional hazard analyses were used to determine its prognostic value for overall survival (OS) and disease free survival (DFS). The Harrell concordance index (C-index), receiver operating characteristic curve (ROC) analysis, and decision curve analysis (DCA) were used to evaluate its role in the improvement of prognostic accuracy of the existing models. Nomogram models containing the SII were then developed and evaluated by R. Patients were divided into low-SII and high-SII groups based on the SII optimal cut-off value 912 calculated by the Youden index in all patients. Higher SII was correlated with more symptoms, longer surgical time, higher WHO/ISUP grade, and longer tumor diameter. Kaplan-Meier analyses revealed significant differences in OS and DFS between two groups. Multivariate analyses revealed that SII was an independent prognostic factor for OS (HR:2.220, p=0.002) and DFS (HR:1.846, p=0.002). Compared with other indicators, SII had a superior accuracy (c-index=0.630 for OS and 0.595 for DFS). It also improved the performance of models for predicting OS and DFS (all p <0.01). Based on the results of LASSO Cox regression analysis, we constructed a nomogram to predict OS and it performed well on both the training cohort (AUC=0.805) and the validation cohort (AUC=0.795). Risk stratification based on nomogram can distinguish patients with different risks (all p <0.001). Preoperative SII is an independent predictive factor for OS and DFS of non-metastatic ccRCC patients with VTT. It can be used to improve the performance of current risk models.

## 1 Introduction

Renal cell carcinoma (RCC) accounts for approximately 3% of all cancers and is the most common solid lesion found in the kidney (1). Venous tumor thrombus (VTT) is observed in about 4-10% of patients with RCC, and the best strategy to these patients is still surgical intervention (2). The prognosis of RCC patients with VTT varies widely, with the 5-year overall survival rate ranging from 34% to 71% (2–4). Several studies have reported the prognostic factors of VTT including tumor size, tumor necrosis, deep invasive tumor thrombus (5), regional lymph node status, distant metastases, and tumor grade (6), etc. However, most of the tumor‐specific factors can only be evaluated by pathological examination after surgery. Therefore, new preoperative predictive markers that are easily applicable should be incorporated into clinical practice.

It is well known that the inflammatory immune response is closely related to the occurrence and progression of cancers. And growing evidence indicates that routine parameters of systemic inflammation including neutrophil, lymphocyte, and platelet counts and their combination, such as neutrophil-to-lymphocyte ratio (NLR) (7), platelet-to-lymphocyte ratio (PLR) (8) are associated with cancer prognosis.

Recently, B. Hu et al. proposed a novel systemic immune-inflammation index (SII) which was defined as platelet counts × neutrophil counts/lymphocyte counts to evaluate the prognosis of hepatocellular carcinoma (9). In recent years, a number of studies have demonstrated its prognostic value in cancers consisting of colorectal cancer, lung cancer, and renal cell carcinoma (10–12). However, previous studies failed to confirm the validity of SII in non-metastatic ccRCC patients with VTT (13–16). In this study, we retrospectively evaluated the survival outcome of non-metastatic ccRCC patients with VTT at three hospitals and assessed the value of the prognostic factors that include SII.

## 2 Materials and methods

### 2.1 Patients selection and cohort definition

In this retrospective study, we collected files of patients diagnosed with non-metastatic ccRCC with VTT in Jinling Hospital, Drum Tower Hospital, and Changhai Hospital between June 2011 and November 2021. In total, 402 RCC patients with VTT were selected among patients with RCC. The exclusion criteria were: a) incomplete clinical data; b) unknown survival time; c) more than one primary tumor; d) not clear cell renal cell carcinoma; e) lymph node or distant metastasis. After exclusion criteria, 328 patients were adopted in this study. Patients were then randomly divided into training (n =213) and validation cohorts (n = 115) with a ratio of 7:3.

### 2.2 Data collection

The following clinical data were collected: the clinicopathological parameters (such as age, gender, symptoms, comorbidity, ECOG Performance Status Scale, TNM stage, Mayo classification, tumor size, WHO/ISUP grade, etc.) and several preoperative blood routine examination results (absolute counts of leukocytes, neutrophils, lymphocytes). All laboratory data were performed within 1 week before surgery. The SII was calculated through the following equation: SII = platelet count × neutrophil count/lymphocyte count. All patients had complete preoperative investigations, including clinical, computed tomography (CT) scan and/or magnetic resonance imaging (MRI) examinations to confirmed the diagnosis. Adjuvant therapy included postoperative adjuvant tyrosine kinase inhibitors and immunotherapy. All patients were regularly followed up after surgery. Overall survival (OS) was defined as the period between the date of surgery and the date of death for all causes or the most recent follow-up. Disease-free survival (DFS) was defined as the time between the date of surgery and the date of recurrence of tumor or death, or last follow-up.

### 2.3 Statistical analysis

Continuous variables are presented as median (IQR) and categorical variables are presented as percentages. Data were tested for normality using the Shapiro-Wilk test. The Student’s t-test was performed to compare continuous variables, and Pearson’s Chi-square test was employed for categorical variables. In case the normal distribution of continuous variables was not assumed, the Mann Whitney U test was performed. Receiver operating characteristic (ROC) curves were plotted for SII to evaluate their sensitivity and specificity. And to determine the optimal cut-off value of inflammatory indices, Youden’s index was used. To evaluate the independent prognostic factors, cox proportional hazards models were utilized for univariate and multivariate analyses. Kaplan-Meier survival curve was plotted to compare the OS and DFS between different groups. Harrell’s C-index was used to measure the predictive accuracy of each model. To further explore clinical utility, we performed a decision curve analysis. To predict patients’ OS, nomogram was created using the rms package in R. Multivariable Cox regression models were trained *via* the least absolute shrinkage and selection operator (Lasso) to determine which variables to include in the nomogram. X-tile (17) was used for survival and relapse risk stratification. All p values were two-tailed, and p<0.05 was considered statistically significant. All statistical analyses were performed by R software (Version 4.1.3).

## 3 Results

### 3.1 Patient characteristics

There were no significant differences in the clinical and pathological characteristics of the training and the validation cohorts. The clinical characteristics of the patients in the training and validation cohorts were summarized in **Table 1**. Among 328 participants, 210 (64.0%) were male and 118 (36.0%) were female. The median age was 61years (IQR 53-68) and 59 (18.0%) of them were older than 70 years. Those who presented with no symptoms were 141 (43.0%) patients. Pathological T stage was T3a in 163 cases (50.0%), T3b in 127 (38.7%), T3c in 26 (7.9%), and T4 in 11 (3.4%). Mayo classification was 0 in 171 patients (52.1%), 56 (17.1%) in I, 58 (17.7%) in II, 22 (6.7%) in III, and 21 (6.4%) in IV. All patients underwent radical nephrectomy with tumor thrombectomy (RNTT), including open RNTT in 168 (51.2%) patients, laparoscopic RNTT in 123 (37.5%), and robot-assisted RNTT in 37 (11.3%) patients. Nearly half (48.2%) of all patients had WHO/ISUP grade III tumors, and 23.8% had grade IV tumors. The maximum diameter of the tumor ranged from 2 to 20 cm with a median size of 7.2 cm (IQR 5.5-9.0). The median follow-up was 24 months (IQR 8-49).

**Table 1:** Patients’ characteristics (n=328).

Patients’ characteristics		All patients	Training set	Validation set	P value
Age (years)					0.177
	<70	269 (82.0%)	170 (79.8%)	99 (86.1%)	
	≥70	59 (18.0%)	43 (20.2%)	16 (13.9%)	
Gender					0.904
	Male	210 (64.0%)	137 (64.3%)	73 (63.5%)	
	Female	118 (36.0%)	76 (35.7%)	42 (36.5%)	
Symptoms					
	Hematuria	114 (34.8%)	75 (35.2%)	39 (33.9%)	0.903
	Pain	72 (22.0%)	48 (22.5%)	24 (20.9%)	0.781
	Other	49 (14.9%)	33 (15.5%)	16 (13.9%)	0.748
	None	141 (43.0%)	88 (41.3%)	53 (46.1%)	0.416
Comorbidity					
	Hypertension	116 (35.4%)	79 (37.1%)	37 (32.2%)	0.399
	Diabetes	49 (14.9%)	31 (14.6%)	18 (15.7%)	0.871
ECOG					0.014
	0	237 (72.3%)	144 (67.6%)	93 (80.9%)	
	≥1	91 (27.7%)	69 (32.4%)	22 (19.1%)	
Hospital stay (days)		13 (9-18)	14 (9-18)	12 (8-17.5)	0.236
Time from diagnosis to surgery (days)		15 (9-33)	15 (8-28)	15 (10-34)	0.213
BMI (kg/m2)		23.50 (21.77-26.10)	23.58 (21.97-26.15)	23.39 (21.57-25.95)	0.279
SII		737.0 (443.5-1176.2)	714 (428-1183)	793 (453-1152)	0.446
Surgical approach					0.209
	Open	168 (51.2%)	102 (47.9%)	66 (57.4%)	
	Laparoscopic	123 (37.5%)	87 (40.8%)	36 (31.3%)	
	Robotic	37 (11.3%)	24 (11.3%)	13 (11.3%)	
Perioperative blood transfusion					0.551
	No	230 (70.1%)	147 (69.0%)	83 (72.2%)	
	Yes	98 (29.9%)	66 (31.0%)	32 (27.8%)	
Surgical time (hours)		4.0 (3.0-5.5)	4.0 (3.0-5.5)	4.0 (3.0-5.5)	0.863
Adjuvant Therapy					0.249
	No	234 (71.3%)	147 (69.0%)	87 (75.7%)	
	Yes	94 (28.7%)	66 (31.0%)	28 (24.3%)	
Tumor side					0.354
	Right	176 (53.7%)	110 (51.6%)	66 (57.4%)	
	Left	152 (46.3%)	103 (48.4%)	49 (42.6%)	
Tumor diameter (cm)		7.2 (5.5-9.0)	7.2 (5.6-9.0)	7.2 (5.0-9.0)	0.48
pT stage					0.73
	3a	164 (50.0%)	111 (52.1%)	53 (46.1%)	
	3b	127 (38.7%)	78 (36.6%)	49 (42.6%)	
	3c	26 (7.9%)	17 (8.0%)	9 (7.8%)	
	4	11 (3.4%)	7 (3.3%)	4 (3.5%)	
Mayo classification					0.47
	0	171 (52.1%)	115 (54.0%)	56 (48.7%)	
	1	56 (17.1%)	36 (16.9%)	20 (17.4%)	
	2	58 (17.7%)	33 (15.5%)	25 (21.7%)	
	3	22 (6.7%)	13 (6.1%)	9 (7.8%)	
	4	21 (6.4%)	16 (7.5%)	5 (4.3%)	
WHO/ISUP grade					0.822
	1	2 (0.6%)	2 (0.9%)	–	
	2	90 (27.4%)	56 (26.3%)	34 (29.6%)	
	3	158 (48.2%)	103 (48.4%)	55 (47.8%)	
	4	78 (23.8%)	52 (24.4%)	26 (22.6%)	
Tumor necrosis					≈1.000
	No	65 (19.8%)	42 (19.7%)	23 (20.0%)	
	Yes	263 (80.2%)	171 (80.3%)	92 (80.0%)	
Follow-up period (months)		24.0 (8.0-49.0)	21.0 (7.0-48.0)	26.0 (10.5-50.0)	0.132
Outcomes					
	Alive	256 (78.0%)	168 (78.9%)	88 (76.5%)	0.675
	Death	72 (22.0%)	45 (21.1%)	27 (23.5%)	
	No recurrence	207 (63.1%)	137 (64.3%)	70 (60.9%)	0.551
	Recurrence	121 (36.9%)	76 (35.7%)	45 (39.1%)	

### 3.2 Determination of optimal cut-off values for SII

According to the optimal cut-off analyzed by using the maximum Youden index, 912 were applied as the optimal cut-off value for SII. The value of the area under the curve (AUC) is calculated to validate it as depicted in **Supplementary Figure 1**.

### 3.3 Association of the SII with clinicopathological parameters

To analyze the association between the SII with clinicopathological parameters, the optimal cut-off of SII was used to dichotomize the 328 patients into high-SII (SII≥912) and low-SII (SII<912) groups. There were 125 (38.2%) patients in the high-SII group and 203 (61.9%) patients in the low-SII group. **Supplementary Table 1** shows that higher SII was significantly correlated with more symptoms (Fever or overt weight loss, p<0.001), longer surgical time (p=0.001), higher WHO/ISUP grade (III-IV)(p<0.001), and longer tumor diameter (p<0.001).

### 3.4 SII signifies a distinct prognosis

**Figure 1:**
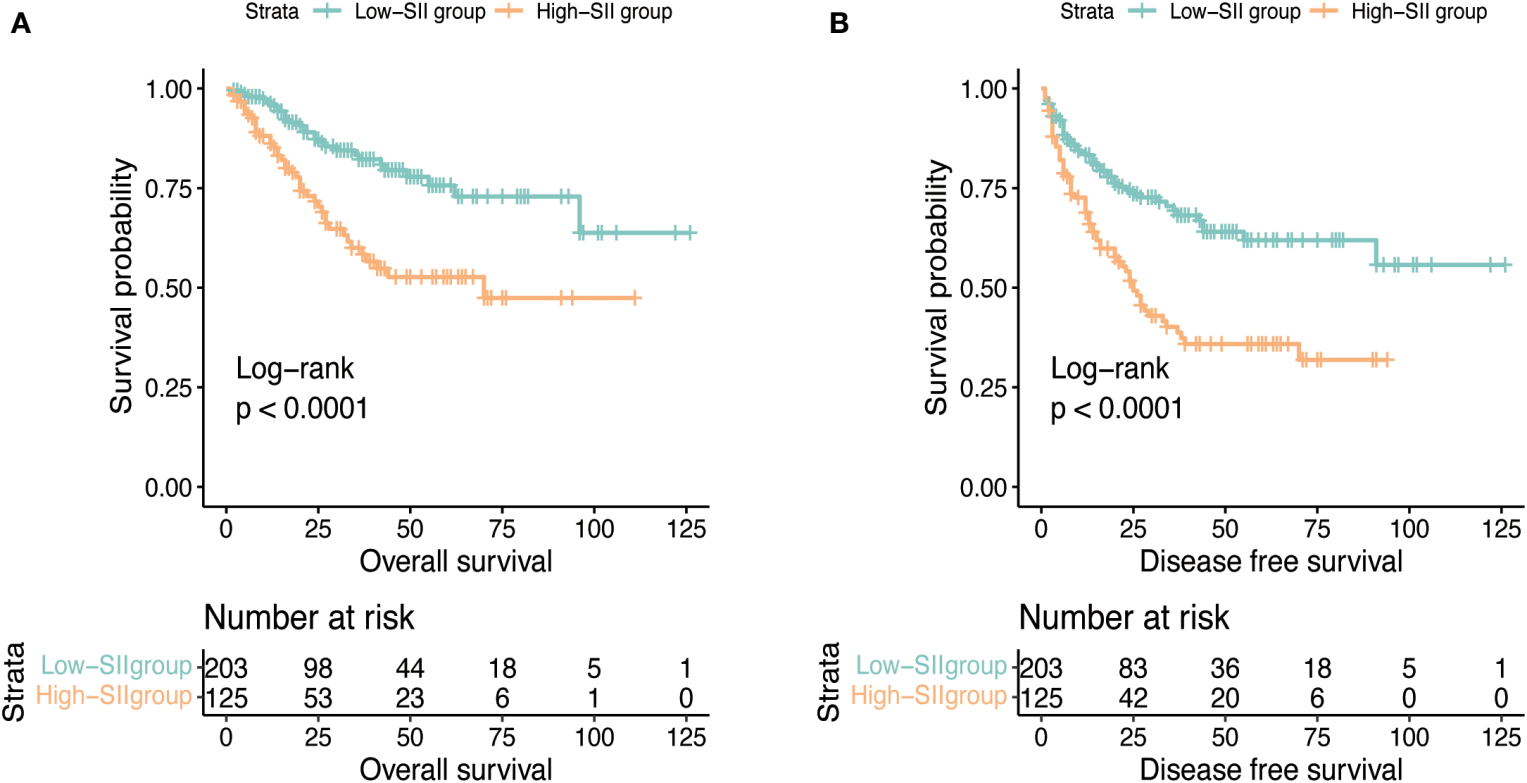
Kaplan-Meier curves for OS **(A)** and DFS **(B)** stratified by SII.

After a median follow-up of 24 months, 121 (36.9%) patients had progressed and 72 (22.0%) patients had died. The median OS was not reached and the median DFS was 70 months (IQR: 38.0-NR). The 1, 3, and 5-year OS was 92.4%, 73.4%, and 66.7% and the 1, 3, and 5-year DFS was 77.7%, 57.5%, and 51.5%. **Figure 1** reflects Kaplan-Meier curves for OS (A) and DFS (B) stratified by SII. The log-rank test estimated a significant difference between SII<912 and ≥912 in OS (p < 0.001) and DFS (p < 0.001). In the high-SII group, the 1, 3, 5-year OS were 86.2%, 60.0% and 52.7% and the 1, 3, 5-year DFS were 68.8%, 40.2% and 35.9%. In the low-SII group, the 1, 3, 5-year OS were 96.2%, 82.2% and 75.7% and the 1, 3, 5-year DFS were 83.3%, 69.3% and 61.9%. **Table 2** shows that pT stage (pT3b: p=0.002; pT3c: p<0.001; pT4: p<0.007), Mayo classification (Level I: p=0.01, Level II: p=0.016; Level III: p=0.005; IV: p<0.001), WHO/ISUP grade 3 (p<0.001), grade 4 (p<0.001), adjuvant therapy (p=0.043), and SII (p<0.001) were significant predictors for OS in univariate analysis. And multivariate analysis revealed that Mayo classification (Level I: p=0.021, Level II: p=0.009; Level III: p=0.043; IV: p<0.001), WHO/ISUP grade 3 (p=0.002), grade 4 (p<0.001), adjuvant therapy (p=0.01), and SII (p=0.002) were independent prognostic factors for OS. And higher SII ≥912 increased the risk of death 2.22 times compared with those in low-SII group. The result of the univariable analysis shown in **Table 3** revealed that BMI (p=0.01), ECOG (P=0.007), surgical approach (Laparoscopic RNTT: p<0.001), pT stage (pT3b: p=0.012; pT3c: p<0.001; pT4: p<0.001), Mayo classification (Level I: p=0.026, Level II: p=0.024; Level III: p=0.07; IV: p<0.001), WHO/ISUP grade 3 (p<0.001), grade 4 (p<0.001), tumor diameter (p=0.016), and SII (p<0.001) were correlated with DFS. Factors that remained significant in multivariable analysis were Mayo IV (p=0.003) thrombus, WHO/ISUP grade 3 (p<0.001), grade 4 (p<0.001), and SII (p=0.001). And patients with higher SII increased the risk of recurrence 1.886 times.

**Table 2:** Univariate and multivariate Cox analyses for OS.

Variables	Univariate analysis		Multivariate analysis	
	HR (95% CI)	p-value	HR (95% CI)	p-value
Gender (Male vs. Female)	1.155 (0.716-1.863)	0.555		
age (≥70 vs. <70)	0.914 (0.501-1.667)	0.768		
BMI (kg/m^2^)	0.934 (0.864-1.010)	0.091		
ECOG (0 vs. ≥1)	2.486 (1.564-3.952)	**<0.001**	1.590 (0.980-2.580)	0.061
Surgical approach				
Open	1 (Referent)			
Laparoscopic	0.640 (0.383-1.068)	0.088		
Robotic	0.797 (0.339-1.874)	0.603		
pT stage				
3a	1 (Referent)			
3b	2.301 (1.343-3.943)	**0.002**		
3c	3.829 (1.842-7.958)	**<0.001**		
4	4.385 (1.501-12.807)	**0.007**		
Mayo classification				
0	1 (Referent)		1 (Referent)	
1	2.348 (1.225-4.500)	**0.010**	2.174 (1.123-4.208)	**0.021**
2	2.268 (1.168-4.404)	**0.016**	2.460 (1.252-4.832)	**0.009**
3	3.062 (1.393-6.730)	**0.005**	2.333 (1.025-5.310)	**0.043**
4	4.619 (2.224-9.595)	**<0.001**	4.279 (2.018-9.071)	**<0.001**
WHO/ISUP grade				
G1+G2	1 (Referent)		1 (Referent)	
G3	5.730 (2.254-14.570)	**<0.001**	4.621 (1.759-12.145)	**0.002**
G4	9.749 (3.739-25.420)	**<0.001**	6.074 (2.230-16.546)	**<0.001**
Adjuvant therapy (No vs. Yes)	0.527 (0.283-0.980)	**0.043**	0.437 (0.232-0.822)	**0.01**
Tumor side (Right vs. Left)	0.944 (0.592-1.504)	0.807		
Tumor diameter (≤7 vs. >7)	1.518 (0.945-2.439)	0.084		
SII (≥912 vs. <912)	2.572 (1.607-4.117)	**<0.001**	2.220 (1.349-3.652)	**0.002**

Bold values represent significant differences ( P < 0.05) in comparison to respective control values.

**Table 3:** Univariate and multivariate Cox analyses for DFS.

Variables	Univariate analysis		Multivariate analysis	
	HR (95% CI)	p-value	HR (95% CI)	p-value
Gender (male vs. female)	1.160 (0.801-1.682)	0.433		
age (≥70 vs. <70)	0.848 (0.525-1.371)	0.502		
BMI (kg/m^2^)	0.923 (0.868-0.981)	**0.010**	0.948 (0.890-1.010)	0.100
ECOG (0 vs. ≥1)	1.654 (1.147-2.385)	**0.007**	0.979 (0.663-1.445)	0.916
Surgical approach				
Open	1 (Referent)		1 (Referent)	
Laparoscopic	0.474 (0.313-0.719)	**<0.001**	0.657 (0.416-1.037)	0.070
Robotic	0.727 (0.386-1.367)	0.322	0.930 (0.482-1.796)	0.829
pT stage				
3a	1 (Referent)			
3b	1.665 (1.119-2.478)	0.012		
3c	2.676 (1.512-4.737)	**<0.001**		
4	4.019 (1.804-8.957)	**<0.001**		
Mayo classification				
0	1 (Referent)		1 (Referent)	
1	1.754 (1.071-2.874)	**0.026**	1.323 (0.791-2.213)	0.287
2	1.756 (1.078-2.861)	**0.024**	1.530 (0.908-2.577)	0.110
3	1.841 (0.952-3.560)	0.070	1.612 (0.801-3.247)	0.181
4	3.132 (1.748-5.613)	**<0.001**	2.634 (1.385-5.007)	**0.003**
WHO/ISUP grade				
G1+G2	1 (Referent)		1 (Referent)	
G3	4.106 (2.222-7.588)	**<0.001**	3.249 (1.714-6.160)	**<0.001**
G4	6.178 (3.231-11.814)	**<0.001**	4.444 (2.249-8.782)	**<0.001**
Adjuvant therapy (No vs. Yes)	1.110 (0.749-1.645)	0.602		
Tumor side (Right vs. Left)	0.752 (0.526-1.075)	0.118		
Tumor diameter (≤7 vs. >7)	1.567 (1.087-2.260)	**0.016**	1.111 (0.759-1.627)	0.588
SII (≥912 vs. <912)	2.223 (1.553-3.181)	**<0.001**	1.886 (1.287-2.763)	**0.001**

Bold values represent significant differences ( P < 0.05) in comparison to respective control values.

Comparisons of the c-index between dichotomized SII (0.630 for OS and 0.595 for DFS) and other indicators including ECOG (0.583 for OS and 0.539 for DFS), tumor diameter (0.586 for OS and 0.565 for DFS), adjuvant therapy (0.561 for OS and 0.523 for DFS), and tumor necrosis (0.551 for OS and 0.524 for DFS) demonstrated the superiority of SII in predicting the outcomes (**Table 4**). The pT stage (0.644 for OS and 0.606 for DFS) and mayo classification (0.652 for OS and 0.602 for DFS) showed no significant difference with SII. The WHO/ISUP grade (0.640 for OS and 0.623 for DFS) were the only indicator better than SII.

**Table 4:** Concordance index analysis of the prognostic accuracy of potential variables.

Variables	Overall survival			Disease free survival		
	C-index	95%CI	p-value	C-index	95%CI	p-value
SII	0.630	0.569-0.691		0.595	0.548-0.642	
ECOG	0.583	0.522-0.644	**<0.001**	0.539	0.496-0.582	**<0.001**
Tumor diameter	0.586	0.512-0.660	**<0.001**	0.565	0.512-0.618	**<0.001**
pT stage	0.644	0.583-0.705	0.293	0.606	0.557-0.655	0.653
Mayo classification	0.652	0.589-0.715	0.226	0.602	0.551-0.653	0.455
Adjuvant Therapy	0.561	0.514-0.608	**<0.001**	0.523	0.478-0.568	**<0.001**
WHO/ISUP grade	0.666	0.605-0.726	**<0.001**	0.645	0.598-0.682	**<0.001**
Tumor necrosis	0.551	0.506-0.596	**<0.001**	0.524	0.485-0.563	**<0.001**

Bold values represent significant differences ( P < 0.05) in comparison to respective control values.

### 3.5 Improved risk stratification by SII

We integrated dichotomous SII into existing models including UISS risk group (18), GRANT score (19), Leibovich model (20), and SSIGN score (21) to see whether it can improve their predictive accuracy. As anticipated, the addition of SII improved models’ performance for predicting OS (0.684 for UISS versus 0.732 for UISS+SII, 0.677 for GRANT versus 0.723 for GRANT+SII, 0.747 for Leibovich versus 0.77 for Leibovich +SII, 0.735 for SSIGN versus 0.765 for SSIGN+SII) and DFS (0.658 for UISS versus 0.692 for UISS+SII, 0.685 for GRANT versus 0.702 for GRANT+SII, 0.692 for Leibovich versus 0.709 for Leibovich +SII, 0.693 for SSIGN versus 0.717 for SSIGN+SII). All the models achieved higher c-indexes than the original models (**Table 5**). It was also confirmed by ROC analysis in **Supplementary Figure 2**. A decision-curve analysis (DCA) shown in **Supplementary Figure 3** demonstrates that the use of the SII in prognostic models contributes added clinical value.

**Table 5:** Improvement of existing model in all patient.

Model	Overall survival			Disease free survival		
	C-index	95%CI	p-value	C-index	95%CI	p
UISS	0.684	0.623-0.745	<0.001	0.658	0.609-0.707	<0.001
UISS+SII	0.732	0.673-0.791		0.692	0.643-0.741	
GRANT	0.677	0.618-0.736	0.002	0.685	0.636-0.734	0.001
GRANT+SII	0.723	0.664-0.782		0.702	0.653-0.751	
Leibovich	0.747	0.688-0.806	<0.001	0.692	0.641-0.743	<0.001
Leibovich+SII	0.77	0.713-0.827		0.709	0.660-0.758	
SSIGN	0.735	0.672-0.798	<0.001	0.693	0.640-0.746	<0.001
SSIGN+SII	0.765	0.704-0.826		0.717	0.666-0.768	

### 3.6 Construction and verification of nomogram

**Figure 2:**
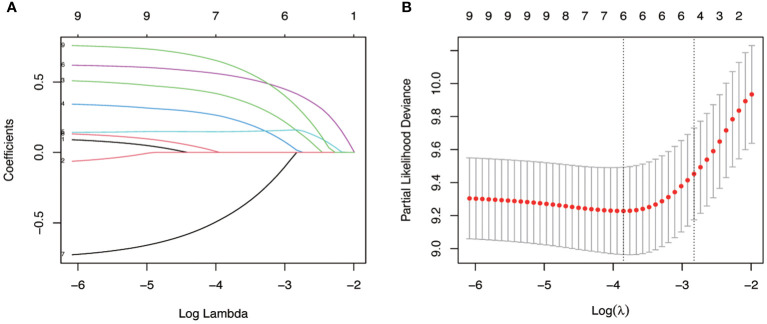
Identification of key variables *via* LASSO cox regression analysis. **(A)** LASSO coefficient profiles of the nine variables for OS. **(B)** Identification of the optimal penalization coefficient lambda (λ=0.078) in the LASSO model with 10-fold cross-validation for OS.

**Figure 3:**
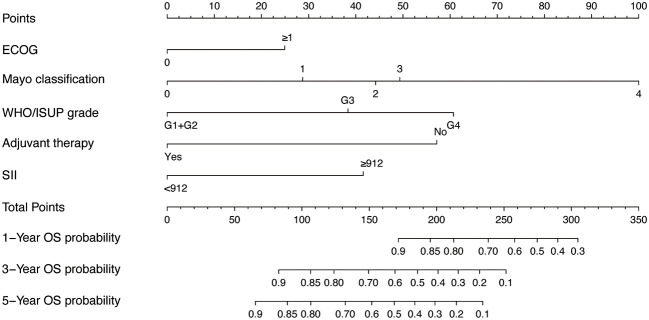
The nomogram for predicting 1-, 3-, and 5-year OS probabilities.

Given the excellent predictive power of SII for OS, to predict survival probability of patients, we constructed a nomogram incorporating the clinical predictive factors and SII. Based on the results of LASSO Cox regression analysis shown in **Figure 2**, five predictive features including ECOG, Mayo classification, WHO/ISUP grade, adjuvant therapy, and SII were selected in the nomogram for OS. Then we developed a nomogram to predict OS in training cohort. **Figure 3** showed the 1-, 3-, and 5-year OS nomogram developed by the Cox proportional hazards results. Time-independent ROC analysis (**Supplementary Figure 4**), DCA (**Supplementary Figure 5**) and calibration curves (**Supplementary Figure 6**) revealed well-powered superiority in prognostic accuracy and clinical value in the training and validation cohorts.

### 3.7 Risk stratification based on the nomogram

**Figure 4:**
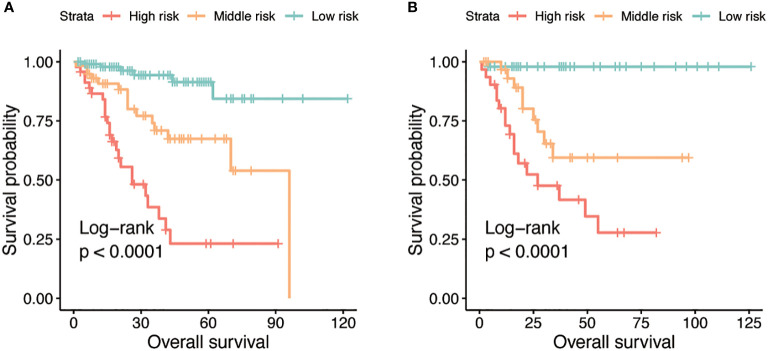
Kaplan-Meier curves for OS stratified by three risk groups in the training **(A)** and validation **(B)** cohorts.

By using X-tile, risk stratifications based on the total points of nomogram were made. The cohorts were divided into three risk groups, with patients in each group having high (total points≥158), middle(108<total points ≤ 158), and low (total points ≤ 108) risk of death, respectively. The Kaplan-Meier OS curves displayed great differences in three groups (**Figure 4**).

## 4 Discussion

VTT is a clinical feature appearing in up to 10% of patients and its relationship with prognosis has been documented (22). TT can lead to inferior vena cava obstruction and progress to liver failure and heart failure. Some of them even develop pulmonary embolism. Hence a clinical model able to predict the prognosis of patients with VTT is a recognized need for cancer research. There have been many reports on the prediction model for RCC, such as the University of California Los Angeles integrated staging system (UISS), GRANT score, Leibovich model, and stage, size, grade, and necrosis (SSIGN) score. There is, however, currently few developed convincing model focusing on indicators of systemic inflammation. In this study, we confirmed that preoperative SII was an independent prognostic factor for non-metastatic ccRCC patients with VTT who underwent surgery. In addition to this, we compared the existing predictive model pertaining to RCC and improved them with SII. We discovered the improved models more suitable for non-metastatic ccRCC patients with VTT. Then, We designed and validated nomogram models to predict OS and DFS in patients.

It’s increasingly recognized that inflammation is inextricably linked to cancer and accumulating evidence indicated that inflammatory response plays a key role in all aspects of the occurrence and progression of cancers. Inflammatory cells can either create favorable conditions for tumor occurrence and growth, favor tumor proliferation and metastasis, or suppress tumors when aggregated. The tumor microenvironment (TME), which contributes to the progression and metastasis of tumors, harbors diverse inflammatory cells including lymphocytes, antigen-presenting cells (macrophages and dendritic cells) as well as mast cells, and the inflammatory mediators they release such as cytokines, chemokines, and transcription factors can drive the inflammatory TME (23).

SII, as a hematological parameter calculated by neutrophil, platelet, and lymphocyte counts, is attracting attention for its simplicity. It has been reported to be a reliable prognostic predictor for various cancers (10–12). Only in the past five years have studies of SII founds its relation to the prognosis of RCC. But researchers mainly concentrated on metastatic RCC (mRCC). As far as we know, it has not been clear whether SII can be used to predict the prognosis of non-metastatic ccRCC patients with VTT.

In terms of the optimal cut-off of SII, previous studies determined different values for RCC. The most commonly used one is 730 having been cited by four papers centered around mRCC treated with surgery or tyrosine kinase inhibitors (TKIs) (13, 15, 16, 24). Other studies also defined the cut-off value of the SII to be 529, 830, and 1291 for Localized RCC or mRCC by the ROC curve or X-tile software (14, 25, 26). Considering there is no study focusing on the cut-off of SII for non-metastatic ccRCC patients with VTT, we selected a cut-off of 912 as the optimal value in the present study by ROC curve.

Univariate Cox regression analysis found Mayo classification and pT stage can predict the death and recurrence. However, due to the similarity between the Mayo classification and the pT stage in patients with VTT, we exclude the latter in multivariable analysis and development of nomogram.

For the first time, we demonstrated that SII is an independent predictive and prognostic factor for non-metastatic ccRCC patients with VTT who underwent surgery. We found that patients in high-SII groups tended to have an inferior OS and DFS compared with patients with lower SII. In this study, multivariate analysis revealed that higher SII was an independent predictor of poor mortality in non-metastatic ccRCC patients with VTT. Furthermore, SII was also an independent predictor of the time to the first recurrence. It is worth noting that surgical approach was not an independent prognostic factor in multivariate analysis. This finding is in good agreement with the results described in previous studies (27, 28). Despite similar oncological outcomes between different surgical approaches, robot-assisted RNTT remains an important approach for its better short-term perioperative outcome including shorter surgical time, less blood loss, lower plasma transfusion volume, lower blood transfusion rate, shorter postoperative hospital stay, and fewer postoperative complications (27–29). And as technology advances (30), the robot-assisted RNTT holds promise for an alternative to traditional open surgery.

There have not been many studies establishing the ability of SII in predicting survival of RCC patients. All of them were focused on the improvement of IMDC Risk Score, which is a prediction model of metastatic RCC. We integrated SII with some other models including UISS risk group, GRANT score, Leibovich model, and SSIGN score. The result corroborated its large contribution to these models to predict both OS and DFS. All this evidence suggests the predictive value of SII. As a result, we suggest that SII should be taken into consideration when building and improving the prediction models of non-metastatic ccRCC patients with VTT. To the best of our best knowledge, no one has yet demonstrated conclusively SII as an independent predictor for OS and DFS in patients with non-metastatic ccRCC with VTT. The performance of nomogram model including SII we then developed can further confirm it.

The mechanisms underlie the association between high SII and poor prognosis of non-metastatic ccRCC patients with VTT remain unclear. Platelets can mediate the extravasation of circulating epithelial tumor cells and maintain or further enhance the extravasation potential of circulating mesenchymal tumor cells (31). Neutrophils can stimulate tumor adhesion and seeding by secreting circulating growth factors (32). Lymphocytes have been proved to exhibit anti-tumor activity by infiltrating into the TME (33).

Study limitations make an overall conclusion about SII extremely difficult. First and foremost, potential bias may exist because of the retrospective nature of our study. Another issue of the current study is that we did not fully explain the specific mechanism of the effect of SII on prognosis. Despite its limitations, this study offers open new scenarios for systemic inflammation in tumor development.

## 5 Conclusions

In summary, our study indicates that high preoperative SII is an independent predictive factor for OS and DFS of non-metastatic ccRCC patients with VTT. It can also be used to improve the performance of existing risk models and should be examined and considered by clinicians for non-metastatic ccRCC patients with VTT. These results may help us to better understand the combined role of neutrophils, platelets, and lymphocytes in cancer and will help elucidate the association between immunity, inflammation and cancer. The results also indicated that, when facing a patient with higher SII, surgeons should not only be cautious in the surgical treatment process, but also fully inform patients to pay attention to its prognosis and give necessary postoperative adjuvant therapy and follow-up management.

## Data availability statement

The raw data supporting the conclusions of this article will be made available by the authors, without undue reservation.

## Ethics statement

The studies involving human participants were reviewed and approved by Ethic Committee of Jinling Hospital. The patients/participants provided their written informed consent to participate in this study.

## Author contributions

YG, YF, XP and YZ collected and analyzed data, obtained the grant, wrote the manuscript. CJ, TZ, HM, HL, JD, JG assisted with data analysis and manuscript preparation. WZ, HG, SG, LQ, and LW assisted with conception and design, material support, data collection and analysis, manuscript review and/or revision. All authors contributed to the article and approved the submitted version.
