# Supplementary material for: Prognostic value of systemic immune-inflammation index in non-metastatic clear cell renal cell carcinoma with tumor thrombus
Source: Front Oncol. 2023 Jan 26;13:1117595. doi: 10.3389/fonc.2023.1117595 (PMC9909392; doi:10.3389/fonc.2023.1117595)
Supplement: Supplementary file 1 [file DataSheet_1.docx]

Supplementary Material

**Supplementary Table 1.** Association of the SII with clinico-pathological parameters in renal cell carcinoma

|  |  | **Low SII(<912)** | **High SII(≥912）** | **p-value** |
| --- | --- | --- | --- | --- |
| No. of patients |  | 203 | 125 |  |
| Age |  | 63.0 (54.0-69.0) | 60.0 (53.0-66.0) | 0.125 |
| Gender |  |  |  | 0.728 |
|  | Male | 128 (63.1%) | 82 (65.6%) |  |
|  | Female | 75 (36.9%) | 43 (34.4%) |  |
| Symptoms |  |  |  |  |
|  | Hematuria | 68 (33.5%) | 46 (36.8%) | 0.624 |
|  | Pain | 38 (18.7%) | 34 (27.2%) | 0.096 |
|  | Other | 17 (8.37%) | 32 (25.6%) | **<0.001** |
|  | None | 96 (47.3%) | 45 (36.0%) | 0.059 |
| Comorbidity |  |  |  |  |
|  | Hypertension | 73 (36.0%) | 43 (34.4%) | 0.866 |
|  | Diabetes | 27 (13.3%) | 22 (17.6%) | 0.367 |
| ECOG |  |  |  | 0.083 |
|  | 0 | 154 (75.9%) | 83 (66.4%) |  |
|  | ≥1 | 49 (24.1%) | 42 (33.6%) |  |
| Surgical approach |  |  |  | 0.718 |
|  | Open | 104 (51.2%) | 64 (51.2%) |  |
|  | Laparoscopic | 74 (36.5%) | 49 (39.2%) |  |
|  | Robotic | 25 (12.3%) | 12 (9.6%) |  |
| Perioperative blood transfusion |  |  |  | 0.51 |
|  | No | 145 (71.4%) | 85 (68.0%) |  |
|  | Yes | 58 (28.6%) | 40 (32.0%) |  |
| Surgical time (hours) |  | 3.5 (3.0-5.0) | 4.5 (3.2-6.0) | **0.001** |
| Tumor side |  |  |  | 0.557 |
|  | Right | 112 (55.2%) | 64 (51.2%) |  |
|  | Left | 91 (44.8%) | 61 (48.8%) |  |
| Tumor diameter (cm) |  | 7.00 (5.00-9.00) | 8.00 (6.50-10.0) | **<0.001** |
| pT stage |  |  |  | 0.158 |
|  | 3a-3b | 177 (87.2%) | 116 (92.8%) |  |
|  | 3c-4 | 26 (12.8%) | 9 (7.20%) |  |
| Mayo classification |  |  |  | 0.517 |
|  | 0 | 111 (54.7%) | 60 (48.0%) |  |
|  | 1 | 31 (15.3%) | 25 (20.0%) |  |
|  | 2 | 34 (16.7%) | 24 (19.2%) |  |
|  | 3 | 12 (5.91%) | 10 (8.00%) |  |
|  | 4 | 15 (7.39%) | 6 (4.80%) |  |
| WHO/ISUP grade |  |  |  | **<0.001** |
|  | 1-2 | 73 (36.0%) | 19 (15.2%) |  |
|  | 3-4 | 130 (64.0%) | 106 (84.8%) |  |
| Tumor necrosis |  |  |  | 0.717 |
|  | No | 42 (20.7%) | 23 (18.4%) |  |
|  | Yes | 161 (79.3%) | 102 (81.6%) |  |

**Supplementary Figure 1.** Receiver operator characteristic analysis area under the curve for the sensitivity and specificity of SII for 1-, 3- and 5-year survival.

**Supplementary Figure 2**. ROC curve analysis for comparing the prognostic accuracy of existing prognostic model with or without SII to predict OS (A, B, C, D) and DFS (E, F, G, H)

**Supplementary Figure 3**. Decision curve analysis showing the net benefit associated with the using of SII comparing with currently reported prognostic models to predict OS (A, B, C, D) and DFS (E, F, G, H)

**Supplementary Figure 4.** Time-ROC curve analysis and area under the curve (AUC) values of nomogram in the training (A) and validation cohorts (B).

**Supplementary Figure 5**. Decision curve analysis showing the net benefit associated with the using of nomogram comparing with currently reported prognostic models to predict OS in the training (A) and validation (B) cohorts

**Supplementary Figure 6.** Calibration curves for 5-year OS in training cohort (A) and validation cohort (B).
